# Supplementary material for: A BET Protein Inhibitor Targeting Mononuclear Myeloid Cells Affects Specific Inflammatory Mediators and Pathways in Crohn’s Disease
Source: Cells. 2022 Sep 12;11(18):2846. doi: 10.3390/cells11182846 (PMC9497176; doi:10.3390/cells11182846)
Supplement: Supplementary file 1 [file cells-11-02846-s001.zip › cells-1795718-supplementary.pdf]

Supplementary Materials

Materials and Methods

Additional Information on Clinical Sampling and Experimental Work-Up:

Intestinal biopsies: biopsies from IBD patients were collected during colonoscopy for assessment of disease activity and/or therapy response (see patient characteristics in Supplementary Table A1: Baseline characteristics IBD patients biopsies). Fresh biopsies (1 or 2 pieces at the time, 2 mm maximum size) were slow frozen in 1 mL of freeze medium (10% dimethyl sulfoxide (DMSO) (Sigma-Aldrich, Burlington, MA, U.S.) and 90% fetal bovine serum (FBS)(Serana, Pessin, Germany), and placed in a Corning CoolCell Cell Freezing Vial Containers (Sigma-Aldrich, Burlington, MA, U.S.) to freeze at a 1°C per minute cooling rate in a -80°C freezer. Samples were stored at -80°C for at least 24 hours and transferred to liquid nitrogen for longterm storage (61). After thawing, a single cell suspension was obtained by incubated the biopsies in digestion medium: collagenase IV C-5138 0.5 mg/ml (Sigma-Aldrich, Burlington, MA, U.S.), DNAse 50 ug/ml (Thermo Fisher Scientific, Waltham U.S.), FBS 2 % (Serana, Germany) in RPMI-1640 medium (Thermo Fisher Scientific, Waltham U.S.) for 45 minutes at 37°C. Biopsies were detached in cells on a 100 µm cell strainer (BD Falcon, Switzerland) placed on a 50-ml tube (Sarstedt, Germany), using the green rubber part of a 2-ml syringe, while rinsing a few times with RPMI-1640 medium (Thermofisher Scientific, Waltham U.S.). The supernatant was then spun at 500 g for 5 min to pellet the cells (61).

PBMCs of vedolizumab treated CD-patients: venous blood was collected during the course of the biological treatment and PBMCs were isolated and cryopreserved according to standard protocol (26, 61). In accordance with the procedure of the intestinal biopsies, PBMCs were placed in a Corning CoolCell Cell Freezing Vial Containers (Sigma-Aldrich, Burlington, MA, U.S.) to freeze at a 1°C per minute cooling rate in a -80°C freezer. Samples were stored at -80°C for at least 24 hours and transferred to liquid nitrogen for longterm storage (61). Patients were categorized as therapy responders or non-responders based a strict combination of endoscopic- (≥50% reduction in SES-CD score) and steroid-free clinical response (≥3 point drop in HBI and HBI ≤4 AND no systemic steroids) and/or biochemical response (≥50% reduction in C-reactive protein (CRP) and fecal calprotectin or a CRP ≤5 g/mL and fecal calprotectin ≤250 µg/g) (2).

Fistula tract tissue of CD patients: CD patients with an active open fistula tract due to their penetrating clinical phenotype underwent surgical intervention (seton placement or drainage of tract) at the Amsterdam UMC, location AMC, the Netherlands. During the surgical intervention, tissue or flush from the tract was obtained. After mechanical digestion, as described in the Material and Methods section, immune cells were isolated according to Ficoll density gradient centrifugation protocol (26). Immune cells obtained from fistulae samples were placed in a Corning CoolCell Cell Freezing Vial Containers (Sigma-Aldrich, Burlington, MA, U.S.) to freeze at a 1°C per minute cooling rate in a -80°C freezer. Samples were stored at -80°C for at least 24 hours and transferred to liquid nitrogen for longterm storage (61).

Reagents.

The following reagents were used in in vitro studies; L-glutamine (Thermo Fisher Scientific, Waltham U.S.), penicillin/streptomycin (Lonza, Switzerland), foetal bovine serum (FBS; Serana, Pessin, Germany), phosphate buffered saline (PBS; Thermofisher Scientific,Waltham U.S.), lipopolysaccharide (LPS Escherichia coli 0111:B4 or Salmonella Typh; Sigma-Aldrich, Burlington, MA, U.S.) and RPMI-1640 medium (Thermofisher Scientific, Waltham U.S.). RPMI-1640 containing 2 mM L-glutamine, 100 U/mL penicillin/streptomycin, and 10% FBS was used for all in vitro cultures.

Mass cytometry analysis

Antibodies; The details of the antibody mass cytometry panel (including clone, metal tag, and supplier) are listed in Supplementary Table A4.

Staining and barcoding; Single cell suspensions were thawed, washed with PBS and incubated with Cell-ID cisplatin to identify dead cells for 5 minutes at room temperature. Cisplatin signal is quenched by washing with Cell Staining Buffer (CSB, Fluidigm, San Francisco, CA, U.S.). Cells were permeabilized by Maxpar Barcode Perm Buffer (Fluidigm, San Francisco, CA, U.S.), incubated with mass tag barcodes in permeabilization buffer, then stained with antibodies against surface targets in the presence of Human TruStain FcX™ Fc Receptor Blocking Solution (Biolegend, San Diego, CA U.S.).

For intracellular staining, cells were washed and incubated with antibodies for intracellular markers (CES1 Polyclonal Antibody, Thermo Fisher Scientific, Waltham U.S.), washed and then stained with a secondary antibody goat-anti-rabbit (polyclonal)-175Lu (Fluidigm, San Francisco, CA, U.S.). CES1 staining in particular was performed with an antibody dilution of 1:800 and 1:200 for the goat-anti-rabbit-175Lu antibody.

After washing with CSB, antibodies were fixed with 1.6% PFA, washed and incubated overnight with 191/193Ir DNA intercalator (1:4000) diluted in Fix-and-Perm Buffer (Fluidigm, San Francisco, CA, U.S.). The next day cells were washed before acquisition on the CyTOF3-Helios.

Mass cytometry data analysis; FCS files were uploaded to R for analysis and quality control. Signal intensities and sample acquisition rates were reviewed for stability over time and events gated based on the condition that flow was stable, excluding calibration beads, and within the 90 % percentile of all Gaussian parameters. Afterwards, $CD45^{+}$ live cells were selected through sequential gating as described before (62). Clusters of phenotypically similar cells were identified using the FlowSOM-package. Initial SOM-clustering was set to 300 clusters, using markers listed in Supplementary Table A4, with the exception of CES1 itself. By excluding CES1 from the calculations, we can make comparisons of the CES1 levels between cell types. The 300 formed clusters were manually metaclustered according to their phenotype lineages. For visualization and cluster interpretation we performed a tSNE dimensionality reduction in R using the same markers as the FlowSOM clustering as input. 50,000 events from each sample were randomly sampled to prevent overcrowding of the tSNE space. Perplexity was set at 30, theta at 0.5 and the number of iterations at 1500. In the case of the PBMC and fistula analysis, a subsequent tSNE projection was performed only on events metaclustered and identified as myeloid cells, using all antibody marker parameters excluding CES1. Different cell types within the myeloid lineage were identified. Different CES1 levels were investigated through median expression levels and percentage of positive cells within the cell subset. Plots were generated using the ggplot2 package (60).

Flow cytometry analysis: cells were washed in PBS and stained for a LiveDead cell viability marker (LifeScience, Amsterdam, The Netherlands). Then cells were stained for surface markers in FACS buffer (0.5% BSA, 0.01% NaN3 in PBS), fixed, permealized and stained for intracellular anti-TNF using Foxp3 / Transcription Factor Staining Buffer Set (eBioscience, San Diego, CA, U.S.). For PBMCs culture; we used the following antibodies; anti-CD3-PerCP (Becton Dickinson, US), anti-CD19-BV421 (Biolegend, US), anti-CD14-PE-Cy7 (Invitrogen), anti-MHCII-AlexaFluor700 (eBioscience, San Diego, CA, U.S.) and anti-TNF-FITC (Invitrogen, US). For ex vivo derived CD fistulae tract cells culture: we used the following antibodies; anti-CD45-PE (eBioscience, San Diego, CA, U.S.), anti-CD3-APC (Biolegend, San Diego, CA U.S.), anti-CD19-BV421(Biolegend, US), anti-CD66b-FITC (eBioscience, San Diego, CA, U.S.), anti-CD14-PE7 (Becton Dickinson, US), anti-MHCII-AF700 (eBioscience, San Diego, CA, U.S.).

Supplementary Figures

A

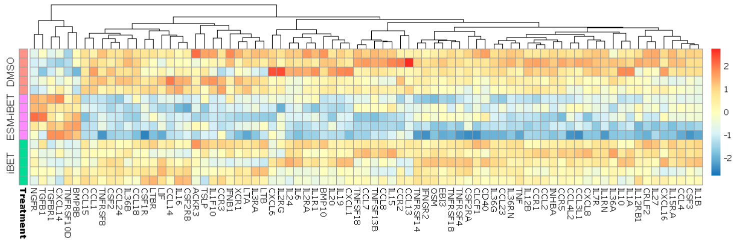

B

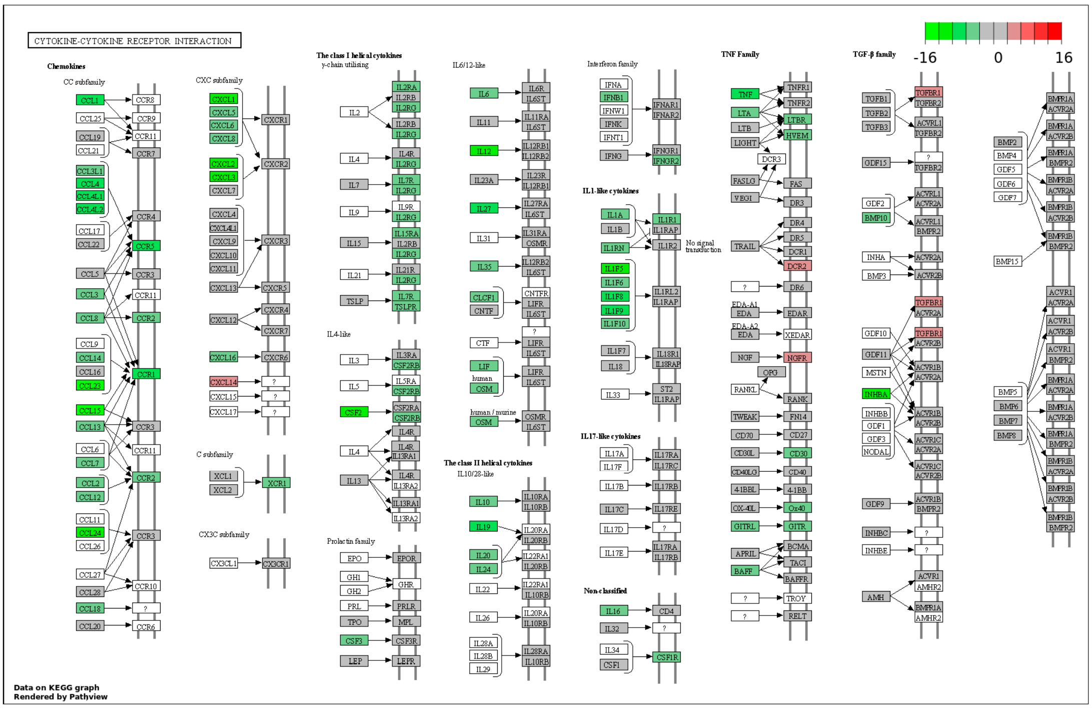

Supplementary Figure S1. ESM-iBET potently modulates multiple cytokines / chemokines transcription in monocytes. (A-B) CD14^{+} monocytes (n = 5 healthy donors) pre-treated (1hr) with DMSO, ESM-iBET (GSK33611910) or iBET (GSK3235220), then stimulated with LPS (4hrs). ESM-iBET (n = 5) is compared to DMSO (n = 5), iBET (n = 5) is shown alongside. (**A**) Heatmap of top differentially expressed genes in the cytokine-cytokine receptor interaction pathway comparing ESM-iBET (n = 5) vs. DMSO (n = 5) pre-treated, LPS stimulated monocytes, iBET pre-treated monocytes (n = 5) are shown alongside. (**B**) The KEGG cytokine-cytokine receptor interaction pathway with colors representing the effect size obtained from interaction analysis. ESM-iBET (n = 5) vs. DMSO (n = 5) pre-treated, LPS stimulated monocytes are compared.

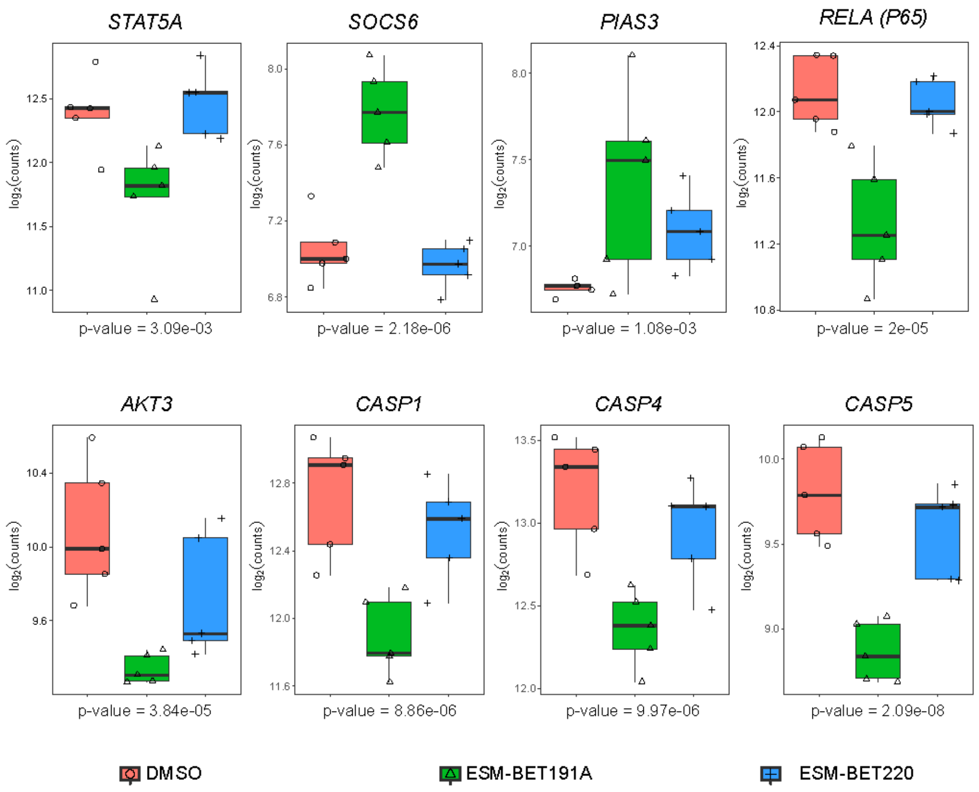

Supplementary Figure S2: ESM-iBET affects transcription of key effector genes in CD within the TNFα, JAK-STAT, NF-kB and NOD2 signaling pathways, with superior potency over iBET. CD14+ monocytes (n = 5 healthy donors) pre-treated (1hr) with DMSO, ESM-iBET (GSK33611910) or iBET (GSK3235220), then stimulated with LPS (4hrs). ESM-iBET (n = 5) is compared to DMSO (n = 5), iBET (n = 5) is shown alongside. Box plots show the expression of selected differentially expressed key effector genes among TNFα, JAK-STAT and NF-kB pathway, expression values are displayed as log2(counts) on the y-axis. In the NF-kB signaling pathway, expression of RELA (P65), a key functional subunit in NF-κB canonical pathway, was found to be downregulated. In PI3K-Akt signaling pathway, AKT3 expression was strongly downregulated. Key effector caspase genes family were targeted with CASP1, CASP4 and CASP5 being efficiently downregulated.

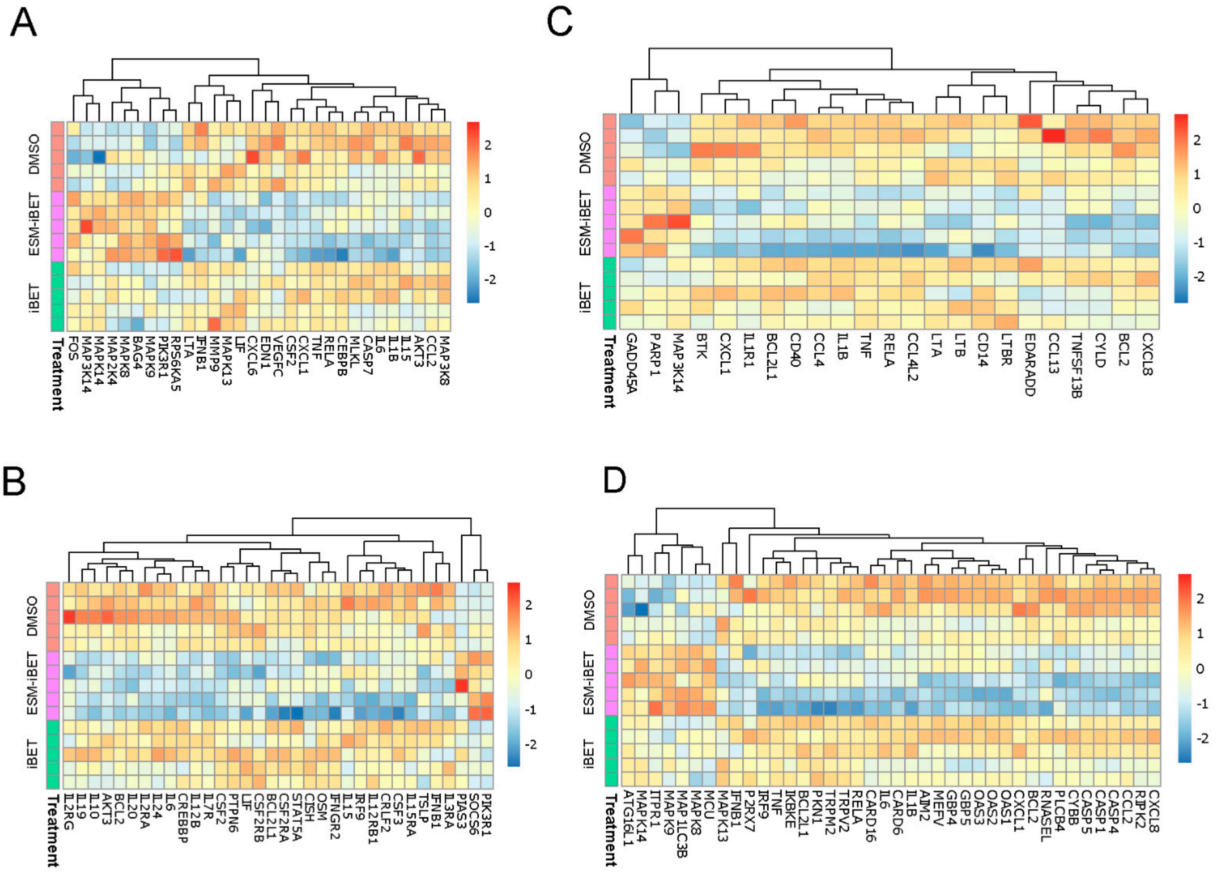

Supplementary Figure S3. ESM-iBET modulate multiple genes within TNFα, JAK-STAT, NF-kB and NOD-like receptor signaling pathways. Heat maps of (**A**) significant 30 differentially expressed genes (DEG) in the TNFα signaling pathway (**B**) 34 DEGs in JAK-STAT signaling pathway (**C**) 23 DEGs in the NF-kB signaling pathway (**D**) and 40 DEGs in the NOD-like receptor signaling pathway, in CD14$^+$ monocytes (n = 5 healthy donors) pre-treated (1hr) with DMSO, ESM-iBET (GSK33611910) or iBET (GSK3235220), then stimulated with LPS (4hrs). ESM-iBET (n = 5) is compared to DMSO (n = 5), iBET (n = 5) is shown alongside.

A

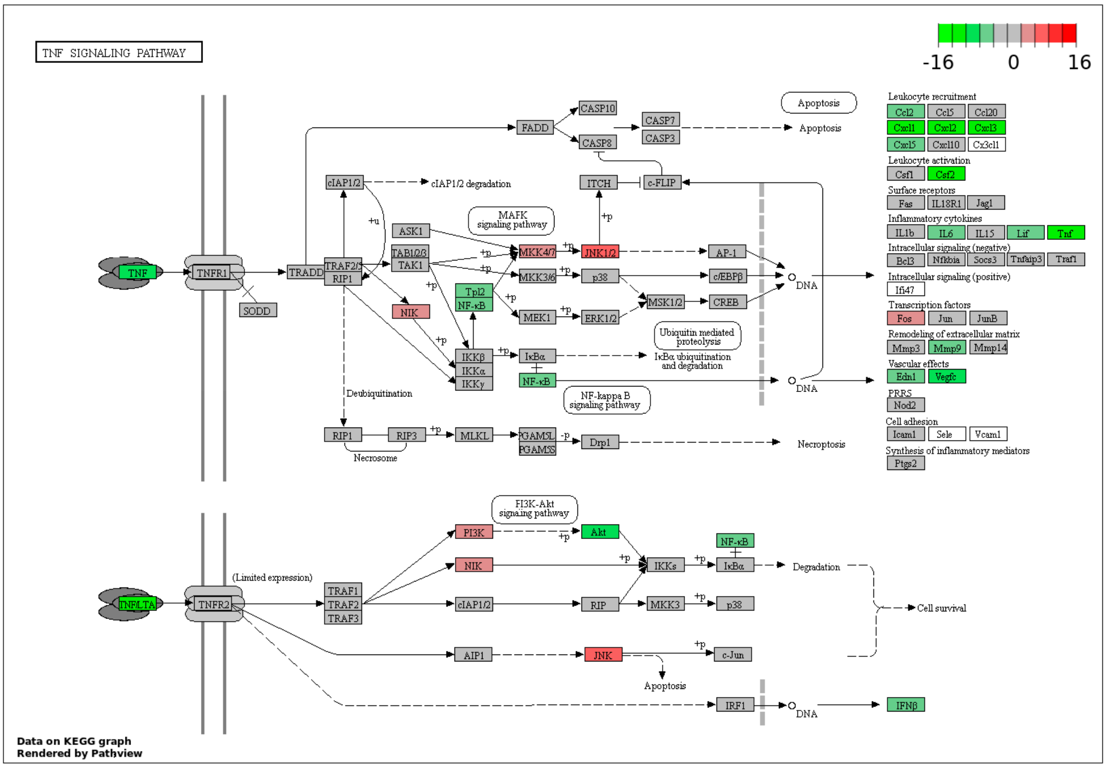

B

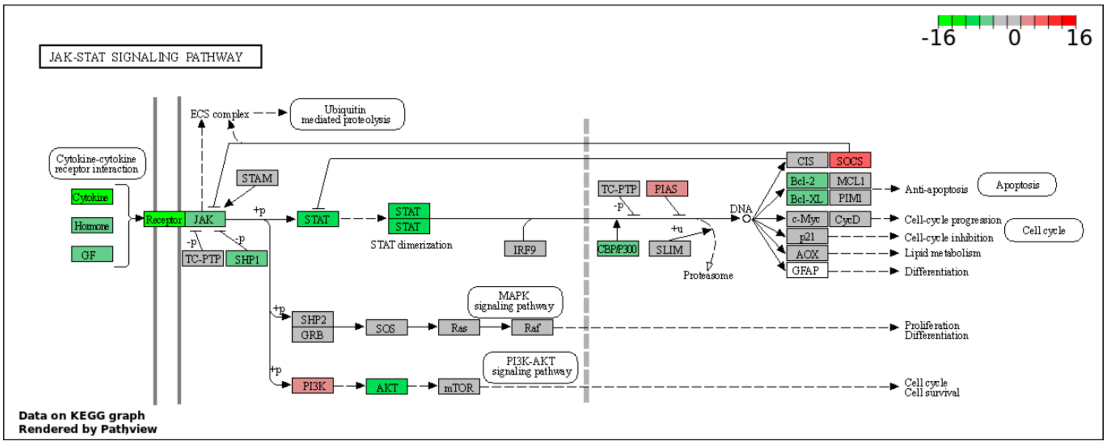

Supplementary Figure S4. The KEGG TNFα and JAK-STAT signaling pathway. (**A**) The KEGG TNFα signaling pathway and (**B**) JAK-STAT receptor interaction pathway with colors representing the effect size obtained from interaction analysis. In CD14$^+$ monocytes (n = 5 healthy donors) pre-treated (1hr) with DMSO, ESM-iBET (GSK33611910) or iBET (GSK3235220), then stimulated with LPS (4hrs). ESM-iBET (n = 5) is compared to DMSO (n = 5), iBET (n = 5) is shown alongside.

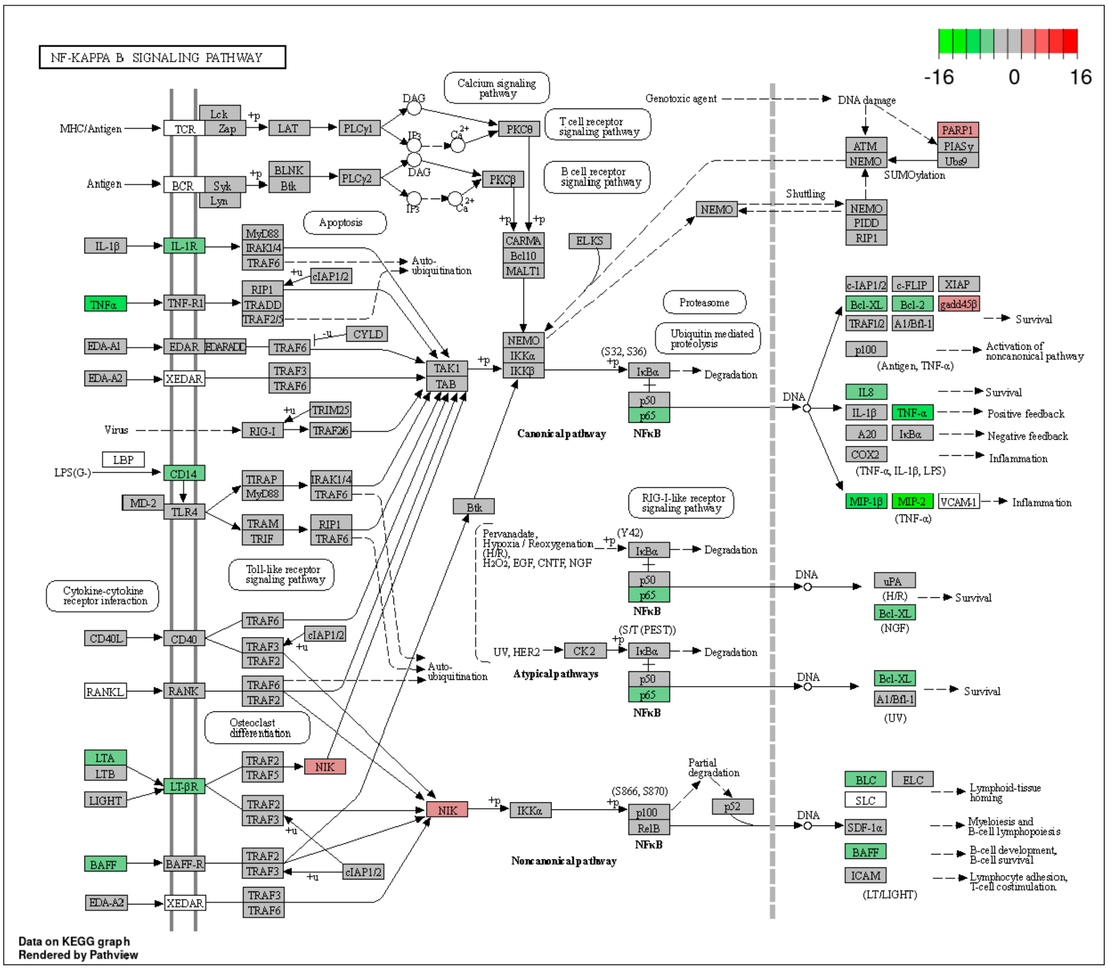

Supplementary Figure S5. The KEGG NF-kB signaling pathway. The KEGG NF-kB signaling pathway with colors representing the effect size obtained from interaction analysis. In CD14+ monocytes (n = 5 healthy donors) pre-treated (1hr) with DMSO, ESM-iBET (GSK33611910) or iBET (GSK3235220), then stimulated with LPS (4hrs). ESM-iBET (n = 5) is compared to DMSO (n = 5), iBET (n = 5) is shown alongside.

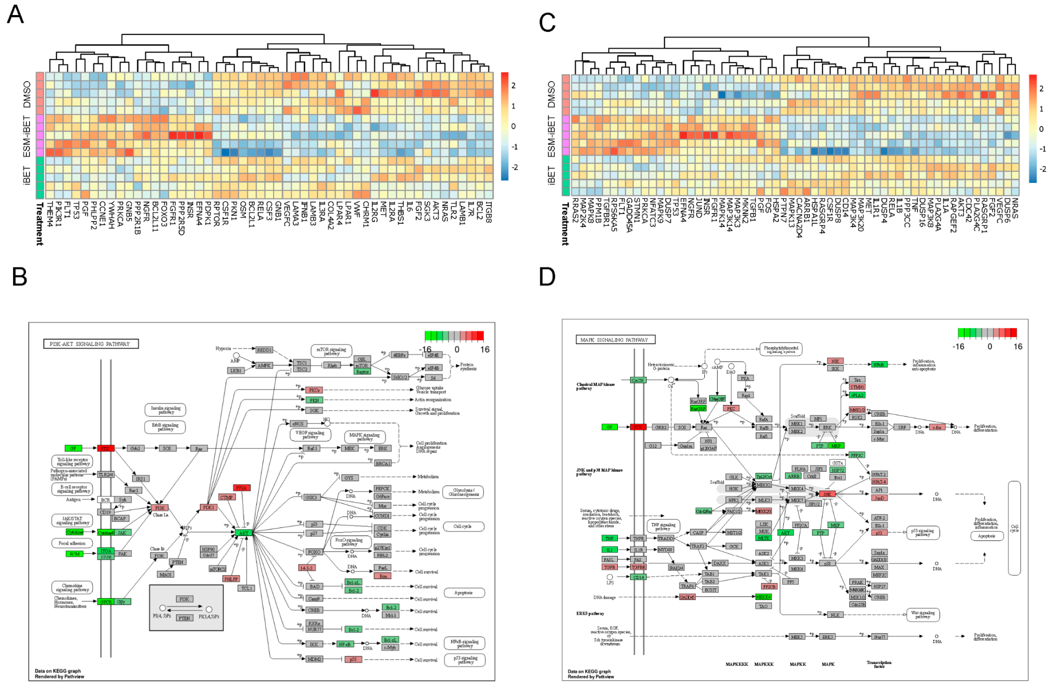

Supplementary Figure S6: ESM-iBET interferes with PI3K-Akt and MAPK signaling pathways via modulating the expression of multiple key effector genes. (**A**–**D**) CD14^{+} monocytes (n = 5 healthy donors) pre-treated (1hr) with DMSO, ESM-iBET (GSK33611910) or iBET (GSK3235220), then stimulated with LPS (4hrs). ESM-iBET (n = 5) is compared to DMSO (n = 5), iBET (n = 5) is shown alongside. Heat map of (**A**) significant 51 differentially expressed genes in the PI3K-Akt signaling pathway together with (**B**) the KEGG PI3K-Akt signaling pathway.Heat map of (**C**) 58 differentially expressed genes in the MAPK signaling pathway together with (**D**) KEGG MAPK signaling pathway. Colors representing the effect size obtained from interaction analysis.

Supplementary tables

Supplementary Table S1. Baseline characteristics IBD patients biopsies.

Baseline Characteristics	IBD Patients Biopsy Retrieved N = 6
**IBD, CD (%)**	3 (50)
Gender, n (%)	
- Female	3 (50)
Age, years, median (IQR)	44 (31.25-55.25)
Disease duration, years, median (IQR)	4 (0.75 - 8)
Disease location in CD, n (%)	
- Ileal disease (L1)	1 (17)
- Colonic disease (L2)	0 (0)
- Ileocolonic disease (L3)	2 (33.3)
- Upper GI involvement (L4)	0 (0)
Disease behavior in CD, n (%)	
- Non structuring non-penetrating (B1)	3 (50)
- Stricturing (B2)	0 (0)
- Penetrating (B3)	0 (0)
- Perianal disease (p)	0 (0)
Disease extension UC (E3, %)	3 (50)
Disease severity UC (S3, %)	1 (17)
Previous IBD related surgery (resection, seton, stricturoplasty), n (%)	3 (50)
Medication, n (%)	
- Immunomodulators	3 (50)
- Prednisone taper scheme	3 (50)
Biological agents, n (%)	0 (0)

Supplementary Table S2. baseline characteristics of Vedolizumab responders and non-responders.

	**Responders (N = 5)**	**Non-Responders (N = 6)**
Female, N (%)	4 (80)	5 (83)
Age, years, median (IQR)	42 (38-44)	58.5 (43-68)
Disease location, n (%)		
- Ileal disease (L1)	3 (60)	3 (50)
- Colonic disease (L2)	1 (20)	-
- Ileocolonic disease (L3)	1 (20)	3 (50)
Disease behavior, N (%)		
- Non structuring/penetrating (B1)	2 (40)	4 (66.6)
- Stricturing (B2)	3 (60)	2 (33.3)
- Penetrating (B3)	0 (0)	0 (0)
- Perianal disease (p)	1 (20)	1 (0.17)
Previous IB- related surgery, N (%)	3 (60)	3 (50)
Concomitant medication, N (%)		
- Immunomodulators	-	-
- Prednisone	-	-

Supplementary Table S3: Baseline characteristics Fistulizing CD patients

**Baseline Characteristics Fistula CD Patients Samples Used for cyTOF and Cell Culture**	**(N = 18)**
Gender, n (%)	
- Female	10 (55.6)
Age, years, median (IQR)	35 (32.25-39)
Disease duration, years, median (IQR)	7.5(3.75-14)
Disease location, n (%)	
- Ileal disease (L1)	4 (22)
- Colonic disease (L2)	5 (28)
- Ileocolonic disease (L3)	9 (50)
- Upper GI involvement (L4)	0 (0)
Disease behavior, n (%)	
- Non structuring non-penetrating (B1)	13 (72)
- Stricturing (B2)	1 (6)
- Penetrating (B3)	4 (22)
- Perianal disease (p)	18 (18)
Previous IBD related surgery (resection, seton, stricturoplasty), n (%)	15 (83)
Medication, n (%)	
- Immunomodulators	4 (22)
- Prednisone taper scheme	0 (0)
Biological agents, n (%)	
-Infliximab	6 (33)
-Adalimumab	4 (22)
- Other (vedolizumab, ustekinumab)	0 (0)
Smoking, n (%)	
- Never	10 (56)
- Active	2 (11)
- Former	3 (17)

Supplementary Table S4: Antibody Panel Mass Cytometry

Antibodies pre-fixation:

Target	Metal/Clone	Source	Panel	Purpose
CD194 (CCR4)	158Gd/L291H4	Fluidigm	PBMC	T and B lymphocytes, basophils, monocytes and NK cells
CD195 (CCR5)	144Nd/NP6G4	Fluidigm	PBMC	T lymphocytes and monocytes
CD183 (CXCR3)	156Gd/G025H7	Fluidigm	PBMC	Chemokine receptor, T lymphocytes
$\alpha4\beta7$	171Yb/-	Takeda	PBMC, fistula tract	Intestinal homing T lymphocytes
CCR9	168Er/L053E8	Fluidigm	PBMC	Intestinal homing T lymphocytes
CCR10	148Nd/314305	R&D	PBMC	Intestinal homing T lymphocytes
Cisplatin	194Pt/-	Fluidigm	Biopsy,PBMC,fistula tract	Live/dead discrimination

Antibodies nuclear staining:

Target	Metal/Clone	Source	Panel	Purpose
CD152 (CTLA-4)	161Dy/14D3	Fluidigm	Biopsy, PBMC, fistula tract	Co inhibitory molecule
αRabbit IgG	175Lu/polyclonal	Fluidigm	Biopsy, PBMC, fistula tract	CES1 Rabbit IgG antibody
CES1	-/polyclonal	Thermo Scientific	Biopsy, PBMC, fistula tract	Myeloid cells

Antibodies post-fixation:

Target	Metal/Clone	Source	Panel	Purpose
CD45	89Y/HI30	Fluidigm	Biopsy, PBMC, fistula tract	All leukocytes
CD49d	141Pr/9F10	Fluidigm	Biopsy, PBMC, fistula tract	Integrin alpha subunit.
CD11a	142Nd/HI111	Fluidigm	Biopsy, PBMC	Integrin alpha L chain
CD19	142Nd/HIB19	Fluidigm	Fistula tract	B lymphocytes
CD5	143Nd/UCHT2	Fluidigm	Biopsy, PBMC	Activated lymphocytes
CD127 (IL7Ra)	143Nd/A019D5	Fluidigm	Fistula tract	Memory and effector T cells, immature B cell proliferation
CD4	145Nd/RPA-T4	Fluidigm	Biopsy, PBMC	T helper lymphocytes

CD68	145Nd/Y182A	Biolegend	Fistula tract	Macrophages
CD8a	146Nd/RPA-T8	Fluidigm	Biopsy, PBMC, fistula tract	Cytoxic T lymphocytes
CD7	147Sm/CD76B7	Fluidigm	Biopsy, PBMC, fistula tract	T lymphocytes
CD66a	148Nd/B1.1/CD66	BD	Fistula tract	Granulocytes
CD25 (IL2R)	149Sm/2A3	Fluidigm	Biopsy, PBMC, fistula tract	Activated T lymphocytes, regulatory T cells
CD134 (OX40)	150Nd/ACT35	Fluidigm	Biopsy, PBMC	Co-stimulatory molecule
CD11c	150Nd/Bu15	Biolegend	Fistula tract	Myeloid cells
CD2	151EuTS1/8/	Fluidigm	Biopsy, PBMC	T lymphocytes, NK cells
CD123	151Eu/6H6	Fluidigm	Fistula tract	Plasmacytoid dendritic cells, basophils
CD95/Fas	152Sm/DX2	Fluidigm	Biopsy, PBMC	Apoptosis
CD141	152Sm/	Fluidigm	Fistula tract	Dendritic cells, myeloid cells
TIM-3	153Eu/F38-2E2	Fluidigm	Biopsy, PBMC	Co inhibitory molecule
CD14	154Sm	Fluidigm	Biopsy, PBMC	Monocytes
CD3	154Sm/UCHT1	Fluidigm	Fistula tract	T lymphocytes
CD279 (PD-1)	155Gd/EH12.2H7	Fluidigm	Biopsy, PBMC, fistula tract	Activated lymphocytes, immune checkpoint
CD163	156Gd/GHI/61	Biolegend	Fistula tract	Macrophages
CD197 (CCR7)	159Tb/GO43H7	Fluidigm	Biopsy, PBMC, fistula tract	Activated lymphocytes, homing to secondary lymphoid organs
CD28	160Gd/CD28.2	Fluidigm	Biopsy, PBMC	Co-stimulatory molecule, activated T lymphocytes
CD14	160Gd/M5E2	Fluidigm	Fistula tract	Monocytes
CD69	162Dy/FN50	Fluidigm	Biopsy, PBMC, fistula tract	Activated lymphocytes
CD206	163Dy/15-2	Biolegend	Fistula tract	Macrophages, dendritic cells
CD161	164Dy/HP-3G10	Fluidigm	Biopsy, PBMC	NK cells
CD43	164Dy/CD43-10G7	Biolegend	Fistula tract	Cell adhesion, T cell costimulation
CD45RO	165Ho/UCHL1	Fluidigm	Biopsy, PBMC, fistula tract	All leukocytes
CD44	166Er/BJ18	Fluidigm	Biopsy, PBMC, fistula tract	Activated lymphocytes
CD27	167Er/O323	Fluidigm	Biopsy, PBMC, fistula tract	Activated lymphocytes
CD45RA	169Tm/HI100	Fluidigm	Biopsy, PBMC	All leukocytes

CD33	169Tm/WM53	Fluidigm	Fistula tract	Myeloid cells
CD3	170Er/UCHT1	Fluidigm	Biopsy, PBMC	T lymphocytes
CD45RA	170Er/HI100	Fluidigm	Fistula tract	All leukocytes
CD57	172Yb/HCD57	Fluidigm	Biopsy, PBMC	NK cells
CD137/4-1BB	173Yb/4B4-1	Fluidigm	Biopsy, PBMC	Macrophages, activated B cells, and dendritic cells
EpCAM	173Yb/	Biolegend	Fistula tract	Epithelial cells
HLA-DR	174Yb/L243	Fluidigm	Biopsy, PBMC, fistula tract	Antigen presenting cells
CD127 (IL7Ra)	176Yb/A019D5	Fluidigm	Biopsy, PBMC	Memory and effector T cells, immature B cell proliferation
CD4	176Yb/RPA-T4	Fluidigm	Fistula tract	T helper lymphocytes
CD16	209Bi/3G8	Fluidigm	Biopsy, PBMC, fistula tract	Monocytes, NK cells, dendritic cells
Barcodes	103–110Pd/-	Fluidigm	Biopsy, PBMC, fistula tract	Staining standardization and doublet discrimination
Iridium	191–193Ir/-	Fluidigm	Biopsy, PBMC, fistula tract	Cell identification
